# Object-Based Dense Matching Method for Maintaining Structure Characteristics of Linear Buildings

**DOI:** 10.3390/s18041035

**Published:** 2018-03-29

**Authors:** Nan Su, Yiming Yan, Mingjie Qiu, Chunhui Zhao, Liguo Wang

**Affiliations:** 1 College of information and communication engineering, Harbin Engineering University, Harbin 150001, China; zhaochunhui@hrbeu.edu.cn (C.Z.); wangliguo@hrbeu.edu.cn (L.W.); 2 School of electronics and information engineering, Harbin Institute of Technology, Harbin 150001, China; xixiqmj@163.com

**Keywords:** dense matching, building objects in urban areas, high-precision disparity, building object structural characteristics

## Abstract

In this paper, we proposed a novel object-based dense matching method specially for the high-precision disparity map of building objects in urban areas, which can maintain accurate object structure characteristics. The proposed framework mainly includes three stages. Firstly, an improved edge line extraction method is proposed for the edge segments to fit closely to building outlines. Secondly, a fusion method is proposed for the outlines under the constraint of straight lines, which can maintain the building structural attribute with parallel or vertical edges, which is very useful for the dense matching method. Finally, we proposed an edge constraint and outline compensation (ECAOC) dense matching method to maintain building object structural characteristics in the disparity map. In the proposed method, the improved edge lines are used to optimize matching search scope and matching template window, and the high-precision building outlines are used to compensate the shape feature of building objects. Our method can greatly increase the matching accuracy of building objects in urban areas, especially at building edges. For the outline extraction experiments, our fusion method verifies the superiority and robustness on panchromatic images of different satellites and different resolutions. For the dense matching experiments, our ECOAC method shows great advantages for matching accuracy of building objects in urban areas compared with three other methods.

## 1. Introduction

With the development of satellite sensors, high-precision 3D reconstruction based on satellite stereoscopic image pairs is one of the most important topics in the remote-sensing field, especially in urban areas. Area-based dense matching is a crucial step in the image-based automatic 3D reconstruction process since feature-based matching cannot provide sufficient information for dense matching [1,2,3]. The dense matching accuracy directly affects the reconstruction results. However, for building objects reconstruction in urban areas, the elevation information is mutated from the building height to zero, and the terrain is discontinuous. Besides, the edge regions of building objects are the nonoverlapping regions, because of satellite stereoscopic image pairs from different angles. They will bring great challenges to area-based dense matching methods leading to obvious matching errors at the elevation discontinuity of building edges. It will be an obstacle to obtain high-precision 3D reconstruction results of building objects in urban areas. The increasing spatial resolution of satellite imaging, ranging from 0.5 m to 2 m in the panchromatic band, has allowed clear edge information to be useful for precise 3D information extraction. In the paper, the building edge characteristics from high-resolution satellite images are fully exploited in the dense matching process. We improved the dense matching method by extracting accurate building edges and outline features for keeping building object structure features in the disparity map.

For the accurate building structure feature extraction, many researchers extract building regions based on image segmentation and classification methods, which explore the full potential of the building features in high-resolution satellite images [4,5]. Karadag et al. [6] integrated various kinds of features related to the buildings to improve the image segmentation process for building extraction. Senaras [7] used a machine learning algorithm to fuse various classifiers trained on the automatically generated dataset with relevant features of buildings for the building classification. Huang and Zhang [8] proposed a morphological building index (MBI) to build the relationship between the morphological operators and the structural features of buildings for building detection. Chaudhuri and Samal [9] exploited the interactions of spatial and spectral domain knowledge about the buildings in the scene to conclude the impact on the results of building segmentation. All the methods mentioned fuse some building characteristics, e.g., shape, texture, shadow, brightness, and local contrast, to distinguish buildings from backgrounds for accurate building extraction. However, the building outlines from the above methods are usually smooth and rounded, which cannot retain the key properties of most buildings with perpendicular straight edges. This might lead to losing the advantage of high resolution. In recent researches, line segments have been considered in the process of building extraction. Wang [10] made the use of line segments extracted and auxiliary vertices in the diagonal by rectangle rule to extract building outlines, which can obtain regular and accurate rectangular building shape. However, for complex and irregular linear buildings, Wang’s method by simple rectangular rule is limited.

For the area-based dense matching methods, there are two main types of algorithms:local methods and global methods. The classical SSD (the Sum of Squared Differences), SAD (Sum of Absolute Differences), NCC (Normal Cross Correlation) all belong to local methods [11]. In the local methods, some feature-based matching constraint methods are proposed to improve the area-based dense matching accuracy [12]. Jyoti Joglekar [13] proposed to use SIFT features and relaxation labeling technique to constraint area-based method. Li [14] proposed key point features to preserve the accurate building shape for matching. Moreover, belief propagation [15], graph cut [16], MRF [17], etc. are all global methods. In most cases, the matching accuracy by global methods is better than that by local methods. However, global methods need cost much more time. Therefore, the semi-global matching (SGM) method [18] is proposed for a tradeoff between accuracy and complexity, which has a great performance in the dense matching field. However, all the matching methods above mainly focus on the global accuracy, which cannot ensure the high matching accuracy for each building object. In the multiview stereo, some object-based methods are proposed such as Y. Furukawa’s method [19] and M. P. Deseilligny’s method [20], which can solve the nonoverlapping problem by multiview observation with more information. They generally need three more images and cannot achieve the accuracy to keep the building object structure characteristics. 

Based on the above review, there are three main contributions in the paper. Firstly, we proposed an improved line adjustment method to correct the edge segments fitting buildings closely. Furthermore, a fusion method based on edge line segments and building detection are proposed to obtain the outlines with accurate building structure characteristics. Finally, but arguably most importantly, edge constraint and outline compensation (ECAOC) dense matching method is proposed in the paper. The improved edge lines are used to optimize matching search scope and matching template window, and the high-precision building outlines are used to compensate the shape feature of building objects. Our ECAOC dense matching method can obtain more accurate matching results to maintain building object structural attribute in the disparity map.

The remaining parts of this paper have been organized as follows. In Section 2, the whole dense matching method algorithm is described. The building structure feature extraction fusion method and edge constraint and outline compensation dense matching method are shown in Section 3 and Section 4 in detail, respectively. Our experimental results are shown by comparing with other methods in Section 5, and the conclusions are drawn in Section 6.

## 2. Proposed Methods 

**Figure 1:**
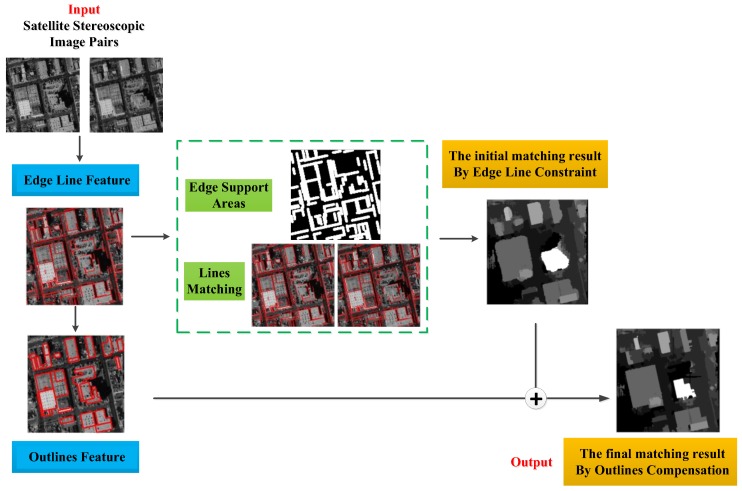
The whole flow chat of the paper.

The whole flow chart of the paper is shown in Figure 1. The proposed object-based dense matching method involves the two main parts: (1) building structure features extraction in the blue box: edge line feature and outline feature; and (2) dense matching method in the yellow box: the initial matching result by the edge line constraint and the final matching result by the outline compensation. In the green dashed box, it is the result of the intermediate process. The proposed dense matching process in the paper is as follows:

**Step** **1.** For edge line extraction, LSD (Line Segment Detector) [21] is employed to obtain basic line detection. In addition, for edge correction, an improved line adjustment method is proposed to correct the edge segments fitting buildings closely.
**Step** **2.** For building outline extraction, a fusion method is proposed to use the edge line feature and the building detection results. The outlines are extracted under the constraint of straight lines for keeping the building structural attribute with parallel or vertical edges.
**Step** **3.** For the initial matching result, an edge constraint dense matching method is proposed. Firstly, line matching results and edge support areas are obtained using the improved edge line feature. Then, line matching results are used to provide more accurate search range and edge support areas improve the matching template window. They both protect the building edge line feature in the area-based dense matching.
**Step** **4.** For the final matching result, the building outline feature are used to compensate the initial matching result for the shape feature of building objects in the post-processing of disparity map.

Therefore, for the whole dense matching process, an edge constraint and outline compensation (ECAOC) dense matching method is proposed to obtain high-precision disparity map of building objects in urban areas. In the following sections, the detail of the algorithm is provided.

## 3. Building Structure Feature Extraction Method

**Figure 2:**
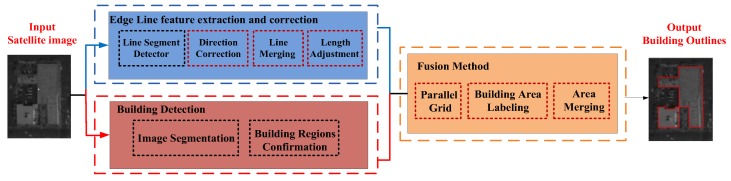
The proposed outline extraction method.

The most important structure features of building objects are edge line feature and outline feature. In addition, the edge line feature extraction is the basic of outline feature extraction. A novel fusion method is proposed to obtain high-precision building outlines automatically from VHR panchromatic satellite images. The proposed algorithm consists of three stages as shown in Figure 2. The main purpose of our fusion method is to combine straight edge line features and building detection results for accurate and regular outline feature with building structural properties.

### 3.1. Edge Line Feature Extraction and Correction

To extract the building outline feature, every straight-line feature in the image is detected first as it represents building edge. LSD is an efficient line detection method proposed by Von Gioi, and with the help of the Number of False Alarms checking. It can give reliable results of line segment detection [21,22].

However, the line extraction results by LSD, which are jumbled and irregular, do not meet the demands of the edge line feature extraction. Therefore, some work [10,23] proposed the joint criteria to improve the edge segments. In this paper, for accurate building outline, we propose an improved line correction method to achieve edge line feature fitting buildings closely, which include three steps: (1) direction correction; (2) line merging; (3) length adjustment.

(1) Direction Correction: Since buildings are mostly modelled as polygonal objects with their edges parallel or perpendicular to each other and buildings in the same area usually share the same orientation. Therefore, edge segments can be corrected to the same or orthogonal to the building orientation [23,24]. To determine the building orientation, the orientation angles of all the line segments, which are the basic lines extracted by LSD, are distributed into *n* bins. In addition, within each bin, the lengths of line segments are accumulated as the value *L*. Suppose *l_i_* is the length of every line segment in the max value *L* corresponding interval and *α_i_* is the angle of the line segment. The building orientation is called primary orientation *α_p_*, which can be computed as (1). As the primary orientation is determined, the secondary orientation, which is approximately perpendicular to the primary orientation, can be obtained in the same way to compute the weighted average. Then, all edge segments that share the same orientation angle, with the primary or secondary orientation angle up to a tolerance, are rotated to be aligned. The tolerance is 22.5 degree in the paper.

(1) $$\alpha_{p}\frac{\sum_{i}\alpha_{i}l_{i}}{\sum_{i}l_{i}}$$

**Figure 3:**
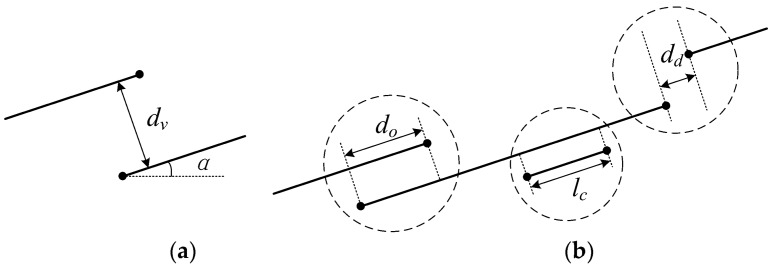
The premise and criteria for merging (**a**) The premise of merging; (**b**) Three criteria of merging (in dashed circles).

(2) Line merging: After the direction correction, the line segments are still just parts of edges and they need to be linked. There is one premise and three criteria for merging procedure, which are shown in Figure 3. Once the premise and one of the criteria are satisfied, the two segments are merged.

- Premise: The lateral distance *d_v_* < *T1*.
- Criterion 1: Two segments are overlapped, and *d_o_* > 0.
- Criterion 2: A short line is covered by another longer segment, and *l_c_* > 0.
- Criterion 3: Two segments are detached, and *d_d_* < *T2*.

If the coordinates of endpoints shown in each dashed circle are (*x*_1_, *y*_1_) and (*x*_2_, *y*_2_), respectively, *d_v_* and *d_d_* can be expressed by the following equations:

(2) $$d_{v}=\left|x_{2}-x_{1}\right|\cdot \sin(\alpha )$$

(3) $$d_{d}=|\left(x_{2}-x_{1})\cdot \cos(\alpha \right)|+|\left(y_{2}-y_{1})\cdot \sin(\alpha \right)|$$

The threshold *T1* and *T2* are discussed in the experimental part. The location of the merged edge segment, which is supposed to be parallel to and close to the longer original segment, is computed by the original lengths as weights.

**Figure 4:**
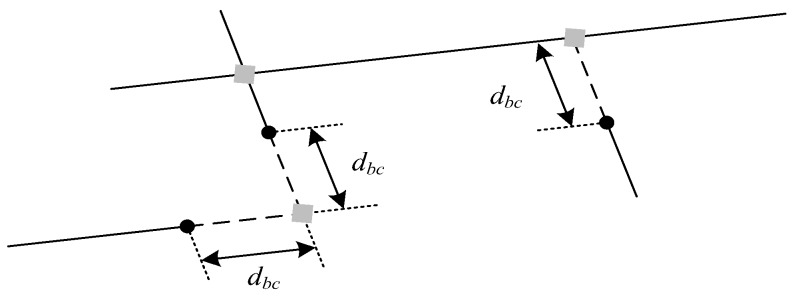
Building corners need to be detected and linked.

(3) Length Adjustment: It is common sense that every building edge should be linked to the building corners. Therefore, the potential building corners are detected, and the corresponding edge segments are extended to be linked to them. Figure 4 shows three kinds of points that need to be detected as building corners and linked under different conditions. The valid line segments represent the edge segments and the dashed line segments represent extending lines of the corresponding segments. All possible building corners and the line endpoints are shown as gray squares and dots, respectively. The three conditions are expressed as:

- Condition 1: The intersection of two edge segments should be detected as a corner.
- Condition 2: The extending lines of two edge segments are intersected, and *d_bc_* < *T3*.
- Condition 3: The extending line of one edge segment is intersected with another edge segment, and *d_bc_* < *T3*.

The threshold *T3* is discussed in the next part. Till now, the building edge segment features are extracted. After the proposed line correction method, the edge segments extracted are neat and regular to fit the building outlines.

### 3.2. Building Detection

For the final building outline feature extraction, building detection is a critical step to confirm the building regions. In the section, a state-of-the-art image segmentation algorithm called ORT is employed. The segmentation method is proposed by McCann [25,26]. It is an unsupervised image segmentation method for our automatic extraction process. Moreover, ORT method does not rely on edge information, which is independent of achieving clustered results for building regions having nothing to do with edge segment features. In this case, the building regions confirmation by ORT can be complementary with the edge segments to be useful for further outline extraction.

Because of the ORT method making full use of the texture and gray-level characteristics, and the building regions are obviously segmented to one or two classes. Therefore, only mean value and standard deviation are used for further building regions confirmation from the few 3–5 clustered results.

### 3.3. The Proposed Fusion Method for Building Outline Feature

Owing to buildings having parallel or vertical edges as the key attribute, we propose a fusion method to extract the building outlines under line constraints based on edge line feature extraction and building detection. The proposed fusion method is divided into the following three steps: (1) parallel grid generation; (2) building area labeling; (3) area merging.

**Figure 5:**
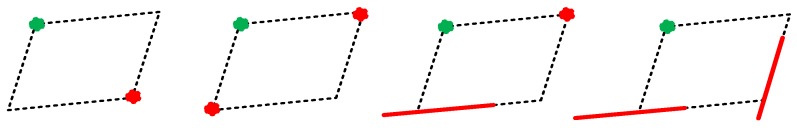
Criteria of parallel grid searching.

(1) Parallel Grid Generation: For the line segments extraction results, assume that the number of lines in the primary and secondary direction is *m* and *n*, respectively. The original image can be simply divided into (*m* + 1) * (*n* + 1) parallel grids by all the edge segments. However, the grids are too small to cause the outline extraction overly to depend on the building detection results. Therefore, not only the edge segments but also the potential building corners are used for parallel grid generation in our method. As is shown in Figure 5, the green dot is a potential building corner as a start point of parallel grid searching. Figure 5 shows four cases to construct a parallel grid by searching along the primary and secondary direction:

- Search only one point: two potential corners in the diagonal of the primary and secondary direction
- Search two points: three potential corners in the primary and secondary direction
- Search one point and one edge segment: two potential corners and one opposite side
- Search two edge segments: one potential corner and two opposite sides

Following our searching criteria, the image is divided into nonoverlapping parallel grids, and the edge segments are preserved completely to solve the excessive segmentation.

**Figure 6:**
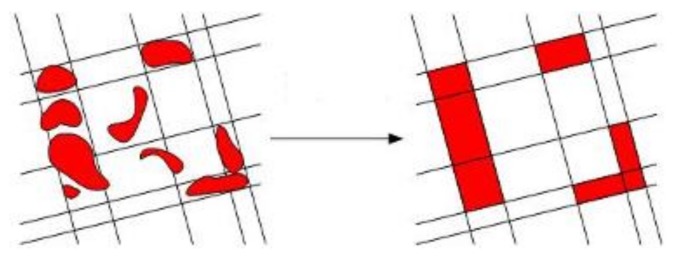
Building area labeling.

**Figure 7:**
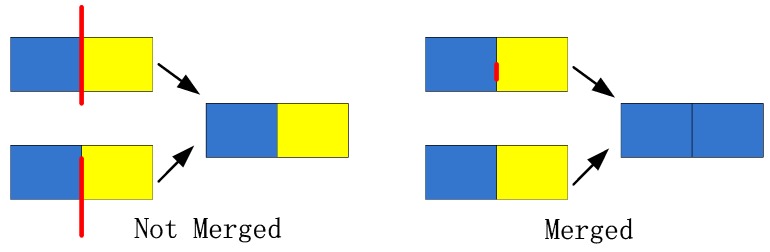
Conditions of building area merged.

(2) Building Area Labelling: After the parallel grid generation, every grid needs to be labelled by the building detection results. The labelling results are computed by the total pixel number of each grid *P_a_* and the building pixel number of each grid *P_b_*. When *P_b_*/*P_a_* > *T4*, the whole grid is labelled building area, as shown in Figure 6.

(3) Area Merging: Although the excessive segmentation problem of parallel grids is considered, there are still two adjacent grids belonging to the same building. As is shown in Figure 7, blue grid and yellow grid represent two adjacent grids and red lines are the edge segments extracted in the previous step. The length of the edge segment is *l* and the length of the grid edge is *l’*. When there is one extracted edge segment between two adjacent grids and *l* > *l’*/2, the two adjacent grids cannot be merged; when there is no extracted edge segment or *l* ≤ *l’*/2, the two adjacent grids should be merged.

## 4. Edge Constraint and Outline Compensation Dense Matching Method

The dense matching of building objects in urban areas is a great challenge. The area-based dense matching methods are all almost based on the continuous disparity assumption in the matching window. However, for building objects in urban areas, the elevation information is mutated from the building height to zero, and the terrain is discontinuous, which lead to obvious matching errors at the elevation discontinuity of building edges. In the paper, we proposed the edge constraint dense matching method by using the improved edge line feature extraction, which can provide more accurate matching search range to decrease the matching errors. Moreover, making use of the accurate outlines, outline compensation matching method is proposed to improve the disparity map for keeping the building structural attribute with parallel or vertical edges.

### 4.1. Edge Constraint Dense Matching Method

For stereoscopic image pairs, dense matching method is carried out on the epipolar image by epipolar constraint, which can make the matching search range reduce from two dimensions to one dimension. In addition to reducing the matching complexity, it also greatly increases the accuracy of matching. However, for building objects in urban areas, the epipolar constraint is not enough to decrease matching errors at the elevation discontinuity of building edges. In the paper, edge constraint dense matching method is proposed to solve the problem of the poor matching accuracy at the building edges, which can use the edge line features to guide the matching search scope and matching template window.

#### 4.1.1. The Optimization of Matching Search Scope 

**Figure 8:**
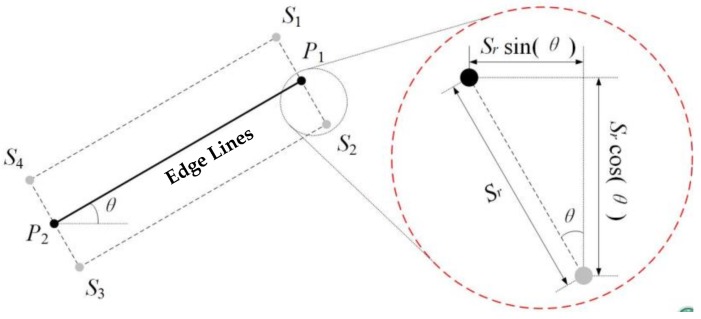
Edge line template window.

For the high-precision dense matching at the building edges, the edge lines are first matched to build the constraint relationship of corresponding matching points. After the edge line extraction by our method, a template window is established for each edge line, as shown in Figure 8. The template window of each edge line is designed to cover the whole edge. Because the width of edge line is only one pixel, a few pixels need be added across the edge line for the template window. By determining the width *Sr*, which is defaulted as 5, the template window can be generated. In Figure 8, the points *P1* and *P2* are endpoints of the edge line and the points *Si* (*i* = 1, 2, 3, 4) are vertexes of the rectangular template window. The details of the part in the small circle are magnified in the red dashed circle on the right to make the clearer explanation. Similar to point matching, edge lines are matched by using the similarity measure criterion. Normalized Cross Correlation (NCC) is employed in the paper.

**Figure 9:**
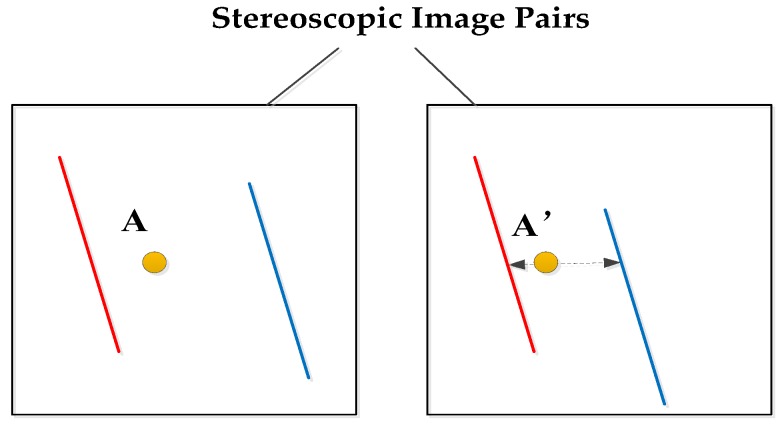
Edge line feature constraint.

Based on the matched edge lines, the points matching search range are narrowed down. When the point is within the two parallel edge lines of buildings, the corresponding matching point is searched for in two matched parallel edge lines of the other image. It is shown in Figure 9. The point A is between the red edge line and the blue edge line in the reference image. The matched A’ is also searched between the adjacent matched red edge line and the matched blue edge line in another image. Moreover, by the epipolar constraint, A’ is searched only on the dashed line with arrows. When the adjacent matched red line and blue line are the two edges of the same building, A’ is searched only on the roof of the building object. When the adjacent matched red line and blue line are the two edges of different buildings or lines of background, A’ is searched only on the street or other background. Our optimization of matching search scope separates the building object and the background to provide more accurate matching search range. 

#### 4.1.2. The Optimization of Matching Template Window 

**Figure 10:**
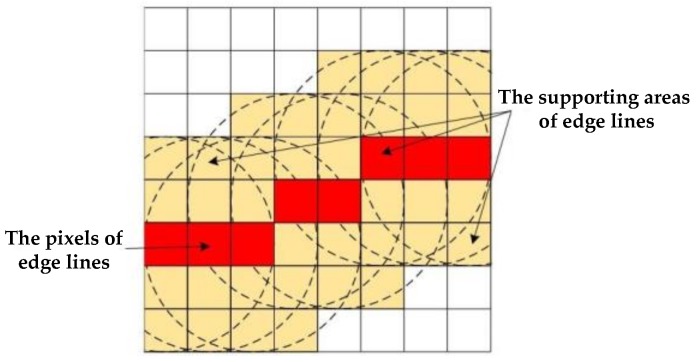
The supporting area of edge line.

**Figure 11:**
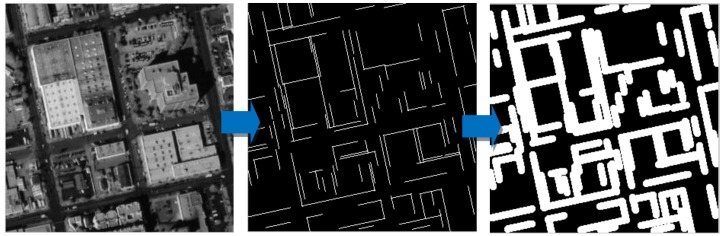
The results of edge line supporting area.

In addition to matching search scope, the size of the matching template window is another important factor of area-based dense matching method. As is well-known, a small template window can protect more detail information of high frequency, while a large template window can make more smooth information. For building objects in urban areas, the building edges obviously need be protected for detail information of high frequency to avoid matching errors. Therefore, making use of edge line extraction results, the supporting area of edge lines is matched by a small template window, and other areas (building roof, street, and other background) are matched by a large template window. The supporting area of edge lines is obtained by morphological dilation, as shown in Figure 10. The red pixels are the edge line with one-pixel width. In addition, yellow area is the supporting area by morphological dilation of edge line. As shown in Figure 11, a small template window is used in the white areas, while a large template window is used in the black areas. The optimization of a matching template window can decrease the matching errors at the elevation discontinuity of building edges to effectively maintain the accurate shape of building objects in the disparity map. 

### 4.2. Outline Compensation Dense Matching Method

**Figure 12:**
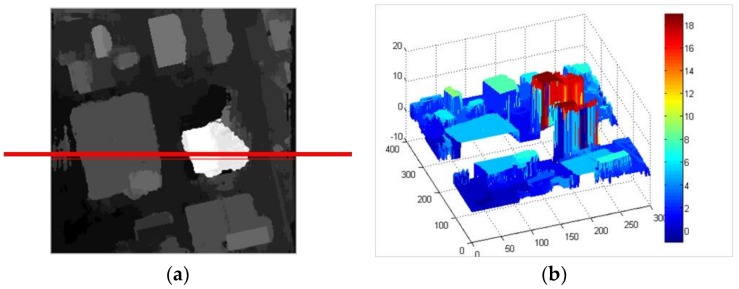

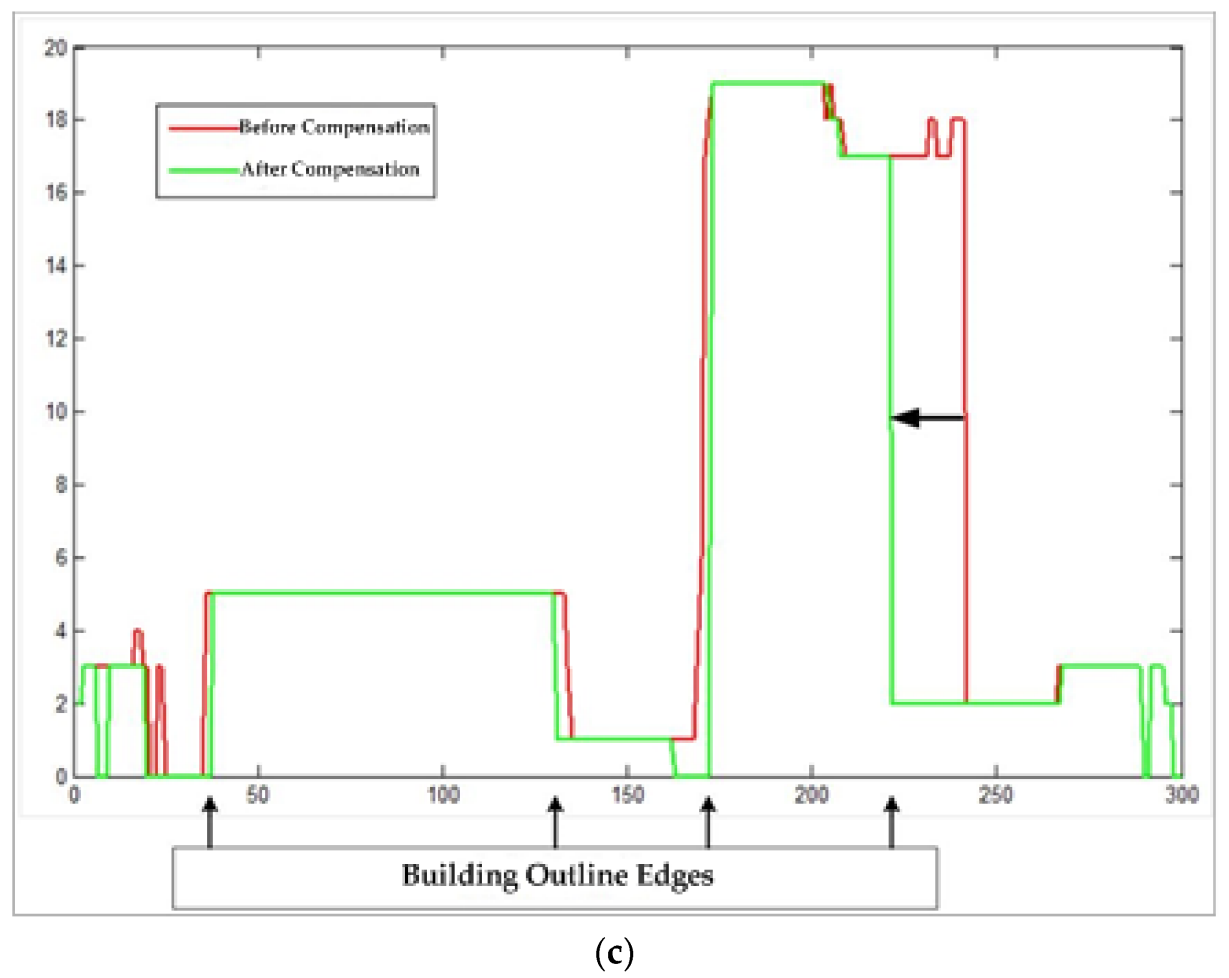
The disparity map compensation by outline feature (**a**) One row of disparity map (**b**) The split stereo display on this row (**c**) The profile optimization of disparity map on this row.

After the edge constraint, the matching errors at the building edges are greatly decreased, but the structure features of building in the disparity map cannot be obtained. The accurate outlines with building structure feature are obtained by our fusion method, which can compensate the disparity map of building objects by post-processing, as shown in Figure 12. Figure 12a is the disparity map after the edge constraint, and the red line represent to select any one row of the disparity map. Figure 12b shows the 3D display of the disparity map, and it is split at the selected row. The process of outline compensation is shown in Figure 12c. In Figure 12c, the red curves are the profile of the 3D disparity map on the selected row, and the green curves are the profile of building outlines at the corresponding position in the 3D disparity map. Because the building outlines by our fusion method are accurate, which can keep the building structure features with parallel or vertical edges. All the building edges of the disparity map after the edge constraint are adjusted the same position with building outline extraction results. In other words, our outline compensation method makes the disparity mutation occur at building edges, which can keep the building structural attribute of the disparity the same as the building outline extraction results. It can further improve the building structural attribute of the disparity, and greatly decrease the matching errors caused by elevation discontinuities at the building edges.

## 5. Experimental Results

### 5.1. Dataset Description

To evaluate the performance of our outline extraction fusion method, four different areas acquired from four different satellites are selected. As shown in Figure 7a–d, in turn, is WorldView-2, QuickBird, IKONOS and GF-1 satellite image. In addition, the resolution is 0.5 m, 0.6 m, 1 m, and 2 m, respectively. In addition, the test images include differently sized and shaped linear buildings. The experiments with different types of satellite data demonstrate the robustness and effectiveness of the proposed algorithm to compare the results by segmentation method and by Wang’s method [11].

To prove the superiority of our ECAOC dense matching method, three different areas of IKONOS stereoscopic image pairs are used. The resolution of satellite images is 1m. In addition, the test images also include differently sized and shaped linear buildings to show the accuracy of building outlines by our fusion method. The experiment results are compared by three other advanced methods. 

### 5.2. Outline Extraction Comparative Experiments

**Figure 13:**
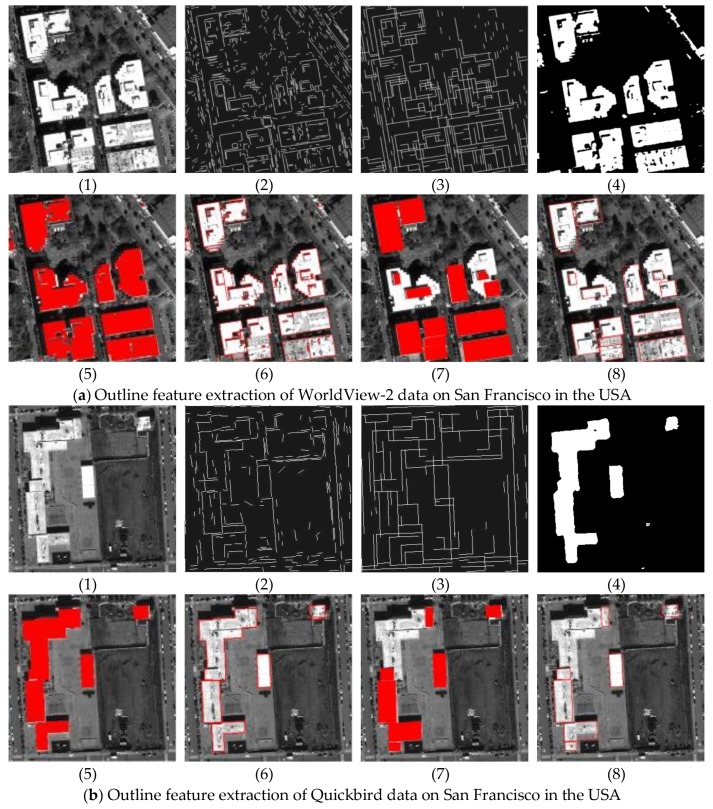

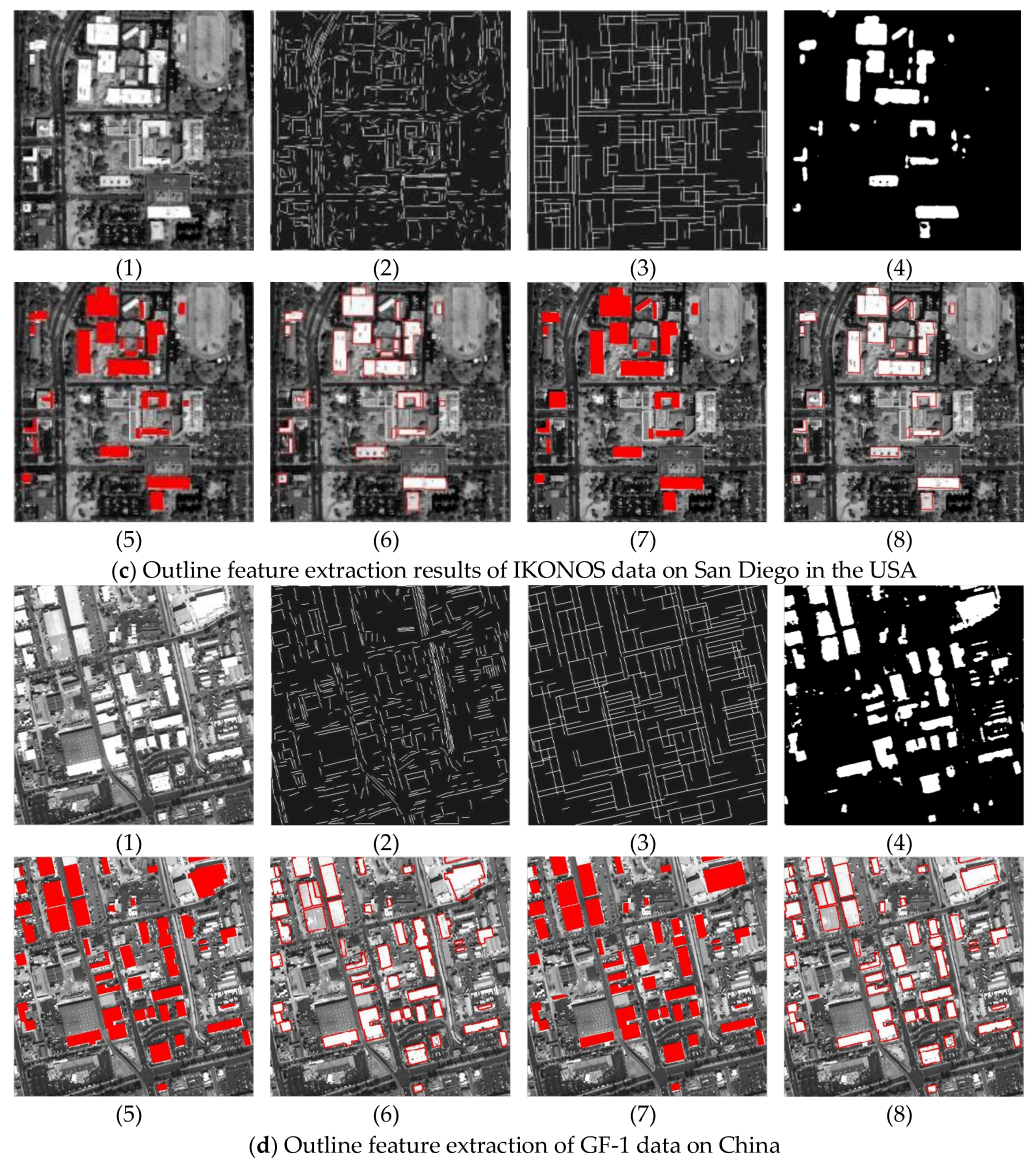
The results of outline extraction and contrast experiment. (**1**) the original satellite image, (**2**) the basic line extraction by LSD (**3**) the edge line feature by our correction method (**4**) the building extraction by segmentation method, (**5**) the building extraction by our method, (**6**) the outline feature by our method, (**7**) the building extraction by Wang’s method, (**8**) the outline feature by Wang’s method.

**Figure 14:**
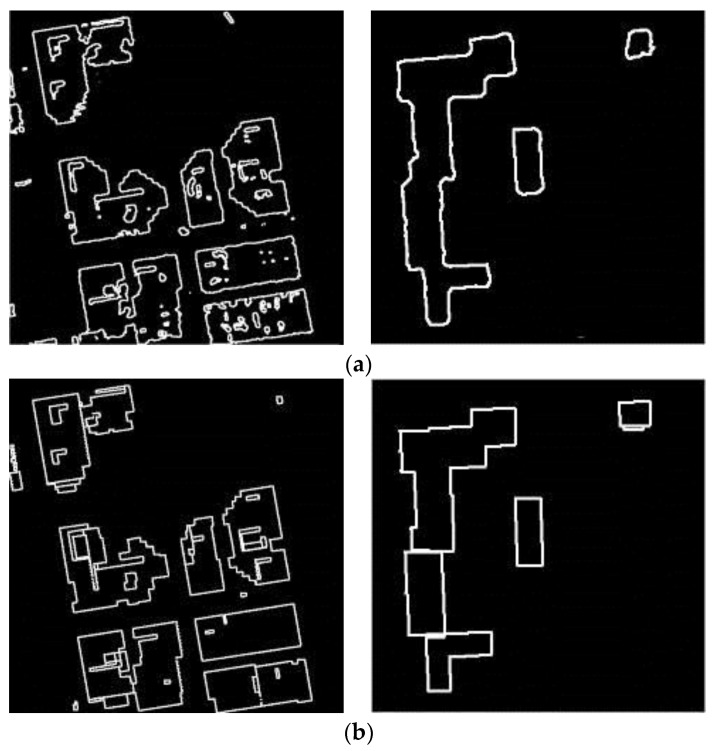
The binary image of the outlines (**a**) outline results by the segmentation method; (**b**) outline results by our method.

In Figure 13, the basic line detection results by LSD and the edge line feature by the proposed correction method are shown in (2) and (3), respectively. The edge line feature by our correction method are obviously closer to the building edges. In addition, (4) is the results of the building extraction by a class of algorithms based on segmentation method, while (5) is building extraction results by our fusion method. The outline is only the extension step of building extraction. Therefore, we first compare the building extraction results between segmentation method (4) and our method (5). The former (4) has a smooth boundary characteristic, while the latter (5) maintains the boundary characteristic of straight lines with perpendicular edges. In Figure 14, the results are the corresponding outlines of Figure 13a,b by segmentation method and our fusion method, respectively. In the enlarged view of outlines (Figure 14), it is obvious that the outlines from our method can better maintain the straight edges of the building structure. Our method can take full advantage of the high resolution to achieve the accurate building shape, which is useful for the building modelling and so on in future work. Moreover, we compare the results of our method with those from Wang’s method. Our method (6) and Wang’s method (8) have similar ideas, both of which make use of straight lines to constrain the outlines for keeping the structural features of the linear buildings. In contrast, two obvious limitations are shown in Wang’s method: (1) undetected auxiliary vertex in the diagonal is needed to close the building boundary, which is limited to detect the rectangle with only four sides; (2) the closed rectangle with low gray value is simply ruled out to be identified as the shadow area, which causes misclassification for the building region. Based on the above analysis, from Figure 13c,d, for the regular rectangle building detection, our results are similar to those from Wang’s method. However, from Figure 13a,b, Wang’s method fails to obtain the outlines for the complex linear building, while our method can still obtain precise and accurate outlines. In short, the proposed fusion method simultaneously uses the advantages of the segmentation method and Wang’s method to obtain better outline results.

It is also worth noting that the experiment Figure 13d is conducted on the GF-1 satellite image with 2 m resolution. For the results, it is obvious that the resolution of the image is lower to influence straight line features. Therefore, 2 m resolution may be the limitation for the high-precision building outline extraction with straight line features.

As mentioned in Section 3, the relevant threshold parameters in different satellite images are shown in Table 1. *T1*, *T2*, and *T3* are the threshold values in the lines correction, and their default values are 2, 5, and 10, respectively. The unit is pixel. The default values are empirical values by experiments. Using the default values can achieve robust results for different satellite images, but the 1 m resolution images have the best performance in our experiments. Therefore, they can be adjusted according to the resolution, as shown in Table 1. In particular, the GF-1 satellite image with 2 m resolution appears to be a blurred area between two neighboring buildings; the premise threshold *T1* still is set as 2 to avoid merging of different buildings. For the percentage of building pixels *T4*, it is 0.7 in the 1 m or higher than 1 m resolution images, while it is 0.5 in the lower than 1 m resolution images.

### 5.3. Dense Matching Comparative Experiments 

**Figure 15:**
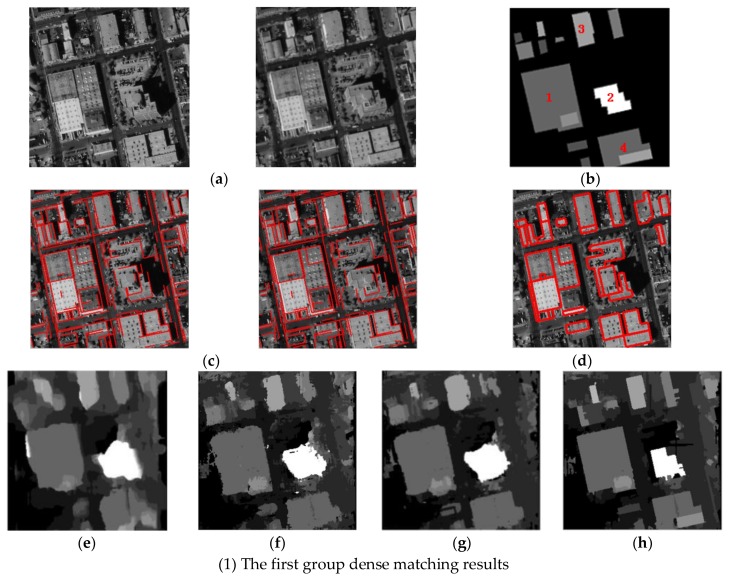

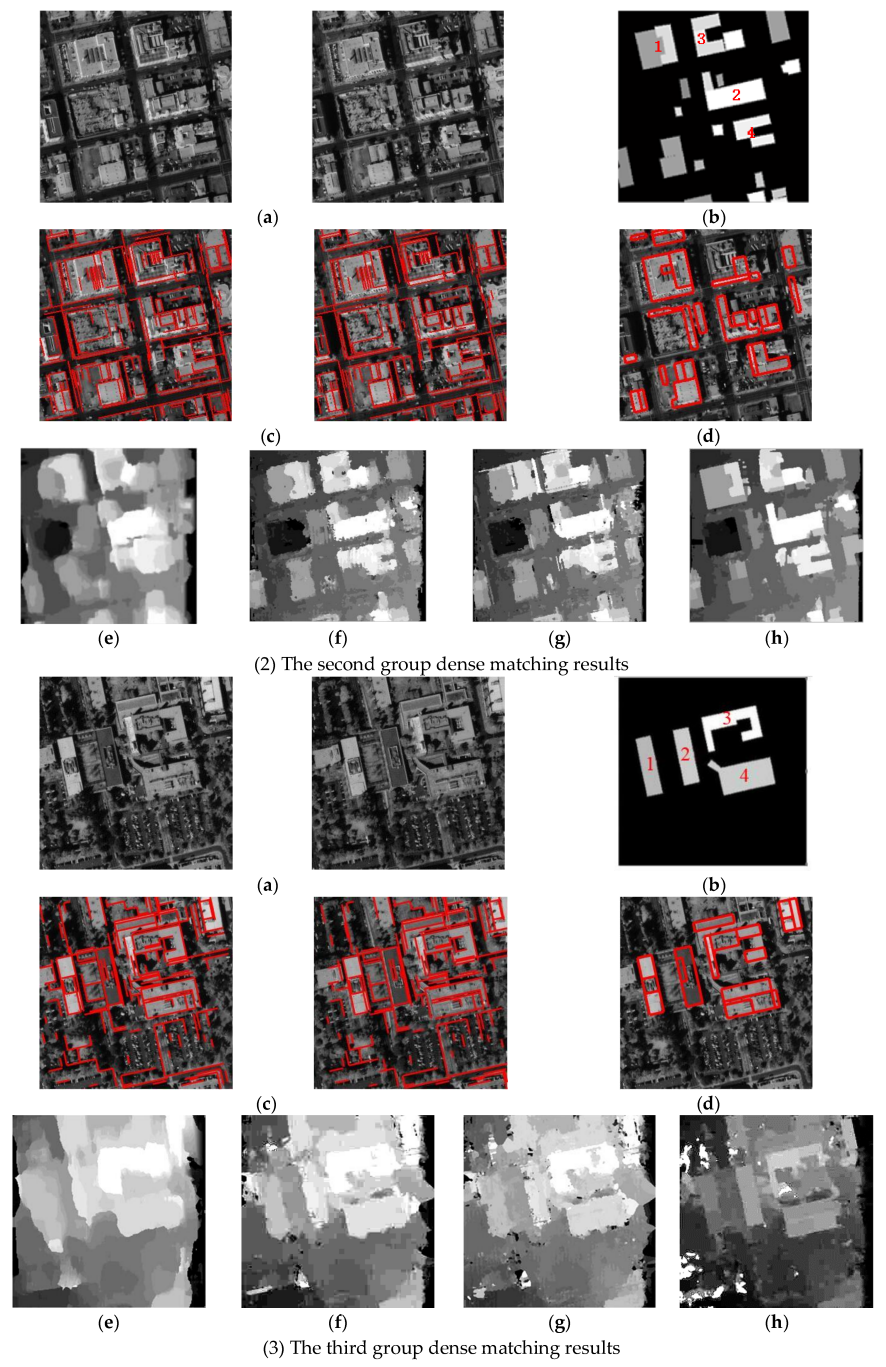
The comparative experiment results of matching disparity map, (**a**) the original stereoscopic image pairs, (**b**) the truth of building objects in the disparity map, (**c**) edge line matching result, (**d**) building outlines by our fusion method, (**e**) matching results by Di’s method [12], (**f**) matching results by J’s method [13], (**g**) matching results by A’s method [18], (**h**) matching results by our ECAOC method.

The comparative experiments of dense matching for stereoscopic image pairs are shown in Figure 15. Figure 15a shows the original stereoscopic image pairs. The corresponding truth of disparity map is made by manual, as shown in Figure 15b. Figure 15c is the lines extraction and lines matching results. The lines matching results accurately build the relationship of the same building edges between the stereoscopic image pairs, which are greatly helpful for the points matching of the building edges. In addition, Figure 15d is the outline extraction of the reference image by our fusion method in the paper. Form the results, the outlines with the accurate building shape are obtained by keeping the structural features of the linear buildings. To prove the superiority of the proposed method for dense matching of stereoscopic image pairs, we compare the matching results by our ECAOC method with the three other methods. Figure 15e–h show the dense matching results by four different methods, which are Di’s method [12], J’s method [13], A’s method [18], and our method in turn. The Di’s matching method is based on the continuous disparity assumption in the matching window to be suitable for continuous terrain in the large scale. However, for building objects in urban area, the elevation information is mutated from the building height to zero, and the terrain is discontinuous, which leads to many apparent matching errors at the elevation discontinuity. From Figure 15e, it is obvious that the matching result incurs many errors at the building edges to miss the structural features of the building objects. From Figure 15f,g, the results are obtained by the J’s method and our A’s method, which are better than Di’s method. J’s method used feature-based matching as a constraint initializing for further dense matching. A’s method is an improved semi-global matching method by H. Hirschmuller. However, both matching results cannot keep the building object structure characteristics in the disparity map. From a subjective perspective, Figure 15h our ECAOC method is obviously the best matching result in the four comparative experiments. In the third group experiments, there are many non-manmade areas such as trees or vegetation in the background, which make the whole disparity map look a little messy. In fact, the building objects in the disparity map still retain clear structure feature by our method. Our edge constraint matching method obtain the accurate disparity map by using the edge lines to separate the building objects and the background, which can provide the more accurate matching search range. The disparity after our outline compensation can maintain the building structural attribute with parallel or vertical edges. It is also closest to the shape of the building objects in the original image. 

**Figure 16:**
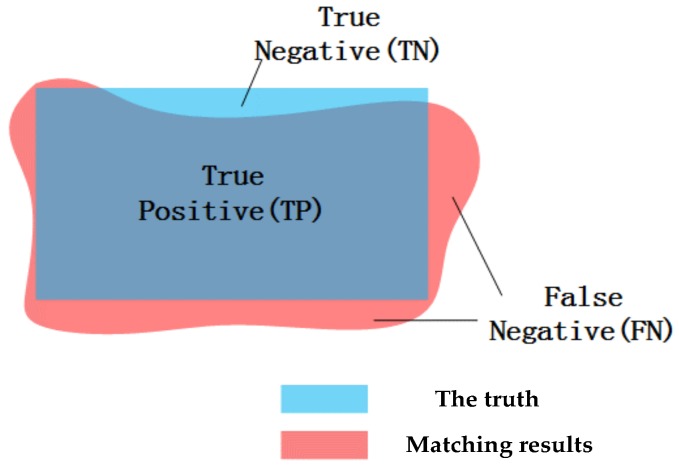
Diagram of object accuracy.

In quantitative evaluation, we proposed the object accuracy to evaluate the matching precision of every object, as shown in Figure 16. The blue area is the truth of the disparity map, while the pink area is the matching results by some methods. The successful matching points in every building object belong to the True Positive, when they meet the following two conditions. (1) The difference between the matching results and the truth of disparity map is less than 1 pixel; (2) The successful matching points of results is within the range of the truth. The points belong to False Negative, when they are in the matching results not in the truth. In addition, the points are True Negative, when they are in the truth not in the matching results. This evaluation index of object accuracy not only describes the matching accuracy of every object but also describes the shape accuracy of the matching results. The object accuracy can be computed as

(4) $$Accuracy=\frac{TP}{TP+TN+FN}\times 100\%$$

where *TP* is the True Positive, *TN* and *FN* represent the True Negative and False Negative, respectively. 

The object accuracy quantitative contrast experiment results are shown in Table 2. From the results, the quantitative evaluation is almost identical with subjective analysis. The matching results by our ECAOC method are the best in the evaluation of all objects. The results of J’s method and A’s method are similar. The Di’s method almost impossible to maintain the shape of any building object. Moreover, the object accuracy of the second group experiments is obviously lower than that of the first and third group. Because the structure features of the building objects in the second group experiments are more complex, which bring greater challenges to all dense matching methods. However, the matching results by the proposed method in the paper are still much better than the three other compared methods. In conclusion, our ECAOC is more effective and superior in the dense matching of building objects. In a way, it can solve the dense matching problem of building objects in the urban terrain discontinuity areas.

## 6. Conclusions 

In this paper, we propose a novel edge constraint and outline compensation (ECAOC) dense matching method to obtain accurate disparity map and keeping building object structure feature. For dense matching of building objects in urban areas, the elevation information is mutated from the building height to zero, and the terrain is discontinuous, which can lead to matching errors at the elevation discontinuity of building edges. Therefore, our work fully analyzes the special terrain of building objects in urban areas, which can greatly increase matching accuracy of building objects by precise building structure feature extraction results. From the outline extraction experimental results, our fusion method obtains clear structure in high-resolution satellite panchromatic images compared with two other methods. From the dense matching experimental results, our ECOAC method proves superior for matching accuracy of building objects in urban areas compared with three other methods. The main contributions in the paper are summarized as follows:

(1) An improved edge line feature correction method is proposed. LSD method is employed to obtain basic line detection. Our method can correct the line segments by LSD to fit building edges closely.
(2) A fusion method is proposed to take advantage of the edge lines and the building areas at the same time, which use straight lines to constrain the building segmentation results for the outlines with a precise shape. Therefore, the outline feature by the proposed method can maintain the building structural attribute with parallel and perpendicular edges, which can be greatly helpful for our dense matching method.
(3) An edge constraint and outline compensation (ECAOC) dense matching method is proposed in the paper. The improved edge lines are used to provide more accurate matching search range, and edge line supporting areas are proposed to improve a matching template window for decreasing the matching errors at the building edges. Furthermore, the high-precision building outlines by our fusion method are used for the final shape compensation of building objects in the disparity map. Our method greatly increases the matching accuracy of building objects in urban areas, especially at the building edges, to obtain the accurate disparity map with clear building structural features.

In conclusion, our outline extraction fusion method and ECAOC dense matching method are proposed especially for dense matching of building objects in urban areas, which can obtain more accurate matching results to maintain building object structural attributes in the disparity map. The high-precision matching disparity map by our method is very helpful for the 3D reconstruction of urban areas in satellite image processing.

## Figures and Tables

**Table 1:** Comparison of the threshold parameters in different satellite resolution images.

	Satellite & Resolution	WorldView-2 0.5 m	QuickBird 0.6 m	IKONOS 1 m	GF1 2 m
Threshold Parameters					
*T1*		4	4	2	2
*T2*		10	10	5	3
*T3*		20	20	10	5
*T4*		0.7	0.7	0.7	0.5

**Table 2:** The object accuracy quantitative contrast experiment results of disparity map.

The First Group				
Object Number	Di’s Method	J’s Method	A’s Method	**Our ECAOC**
1	0.603	0.880	0.868	**0.934**
2	0.286	0.499	0.463	**0.742**
3	0.242	0.665	0.660	**0.869**
4	0.552	0.690	0.684	**0.837**
Average	0.421	0.684	0.669	**0.846**
The Second Group				
Object Number	Di’s Method	J’s Method	A’s Method	**Our ECAOC**
1	0.379	0.558	0.633	**0.744**
2	0.369	0.542	0.602	**0.776**
3	0.126	0.513	0.536	**0.657**
4	0.300	0.439	0.533	**0.623**
Average	0.293	0.513	0.576	**0.700**
The Third Group				
Object Number	Di’s Method	J’s Method	A’s Method	**Our ECAOC**
1	0.435	0.633	0.652	**0.889**
2	0.403	0.534	0.523	**0.903**
3	0.489	0.505	0.511	**0.763**
4	0.613	0.715	0.761	**0.897**
Average	0.407	0.597	0.611	**0.864**
